# Supplementary material for: Modeling glioblastoma heterogeneity as a dynamic network of cell states
Source: Mol Syst Biol. 2021 Sep 16;17(9):e10105. doi: 10.15252/msb.202010105 (PMC8444284; doi:10.15252/msb.202010105)
Supplement: Supplementary file 5 — Source Data for Figure 3 [file MSB-17-e10105-s001.zip › Figure3A_sourcedata/GSEA_3065/nosigdb_state5.GseaPreranked.1622718435914/NB_KI_CLUSTER1.html]

Details for gene set NB\_KI\_CLUSTER1[GSEA]

|  || Dataset | state5 |
| Phenotype | NoPhenotypeAvailable |
| Upregulated in class | na\_neg |
| GeneSet | NB\_KI\_CLUSTER1 |
| Enrichment Score (ES) | -0.9562667 |
| Normalized Enrichment Score (NES) | -2.7098033 |
| Nominal p-value | 0.0 |
| FDR q-value | 0.0 |
| FWER p-Value | 0.0 |
Table: GSEA Results Summary

  

Fig 1: Enrichment plot: NB\_KI\_CLUSTER1      
 Profile of the Running ES Score & Positions of GeneSet Members on the Rank Ordered List

  

| PROBE | GENE SYMBOL | GENE\_TITLE | RANK IN GENE LIST | RANK METRIC SCORE | RUNNING ES | CORE ENRICHMENT || 1 | RPS29 |  |  | 8534 | -0.448 | -0.9363 | Yes |
| 2 | RPL13A |  |  | 8714 | -0.578 | -0.9345 | Yes |
| 3 | RPS7 |  |  | 8791 | -0.644 | -0.9186 | Yes |
| 4 | RPL21 |  |  | 8802 | -0.655 | -0.8950 | Yes |
| 5 | RPL10 |  |  | 8835 | -0.690 | -0.8726 | Yes |
| 6 | RPL27A |  |  | 8838 | -0.696 | -0.8465 | Yes |
| 7 | RPS27 |  |  | 8865 | -0.727 | -0.8219 | Yes |
| 8 | RPS27A |  |  | 8875 | -0.742 | -0.7949 | Yes |
| 9 | RPL34 |  |  | 8877 | -0.744 | -0.7669 | Yes |
| 10 | RPL39 |  |  | 8893 | -0.763 | -0.7398 | Yes |
| 11 | RPS25 |  |  | 8909 | -0.787 | -0.7117 | Yes |
| 12 | RPL3 |  |  | 8916 | -0.803 | -0.6821 | Yes |
| 13 | RPS4X |  |  | 8923 | -0.826 | -0.6516 | Yes |
| 14 | RPS18 |  |  | 8926 | -0.829 | -0.6205 | Yes |
| 15 | RPS6 |  |  | 8929 | -0.837 | -0.5891 | Yes |
| 16 | RPLP2 |  |  | 8932 | -0.844 | -0.5575 | Yes |
| 17 | RPL23A |  |  | 8934 | -0.845 | -0.5257 | Yes |
| 18 | RPS3 |  |  | 8936 | -0.850 | -0.4937 | Yes |
| 19 | RPS19 |  |  | 8941 | -0.855 | -0.4619 | Yes |
| 20 | RPL13 |  |  | 8944 | -0.862 | -0.4296 | Yes |
| 21 | RPS15A |  |  | 8947 | -0.870 | -0.3969 | Yes |
| 22 | RPL32 |  |  | 8953 | -0.891 | -0.3639 | Yes |
| 23 | RPS3A |  |  | 8957 | -0.906 | -0.3300 | Yes |
| 24 | EEF1A1 |  |  | 8958 | -0.915 | -0.2954 | Yes |
| 25 | RPS2 |  |  | 8960 | -0.923 | -0.2607 | Yes |
| 26 | TPT1 |  |  | 8967 | -0.951 | -0.2255 | Yes |
| 27 | RPLP1 |  |  | 8968 | -0.958 | -0.1893 | Yes |
| 28 | RPL18A |  |  | 8969 | -0.964 | -0.1529 | Yes |
| 29 | RPL28 |  |  | 8972 | -0.993 | -0.1157 | Yes |
| 30 | RPL41 |  |  | 8974 | -1.013 | -0.0775 | Yes |
| 31 | RPS12 |  |  | 8975 | -1.034 | -0.0385 | Yes |
| 32 | RPS8 |  |  | 8976 | -1.044 | 0.0009 | Yes |
Table: GSEA details [plain text format]

  

Fig 2: NB\_KI\_CLUSTER1: Random ES distribution      
 Gene set null distribution of ES for **NB\_KI\_CLUSTER1**
